# Does high muscle temperature accentuate skeletal muscle injury from eccentric exercise?

**DOI:** 10.14814/phy2.12777

**Published:** 2016-05-15

**Authors:** John W. Castellani, Edward J. Zambraski, Michael N. Sawka, Maria L. Urso

**Affiliations:** ^1^ U.S. Army Research Institute of Environmental MedicineNatickMassachusetts

**Keywords:** Cytokines, eccentric exercise, heat‐shock proteins, interleukins, maximal voluntary contraction

## Abstract

Hyperthermia is suspected of accentuating skeletal muscle injury from novel exercise, but this has not been well studied. This study examined if high muscle temperatures alters skeletal muscle injury induced by eccentric exercise (ECC). Eight volunteers (age, 22.5 ± 4.1 year; height, 169.5 ± 10.8 cm; body mass, 76.2 ± 12.6 kg), serving as their own control, and who were not heat acclimatized, completed two elbow flexor ECC trials; in one trial the biceps were heated >40°C (HEAT) and in the other trial there was no heating (NON). HEAT was applied with shortwave diathermy (100 W) for 15 min immediately before the first ECC bout and for 2 min in between each bout. Individuals were followed for 10 days after each ECC session, with a 6‐week washout period between arms. The maximal voluntary isometric contraction decreased by 41 ± 17% and 46 ± 20% in the NON and HEAT trials, respectively. Bicep circumference increased by 0.07 ± 0.08 mm (4%, *P* = 0.04) and relaxed range of motion decreased by 11.5 ± 8.2° (30%, *P* < 0.001) in both trials. Serum creatine kinase peaked 72‐h following ECC (NON: 6289 ± 10407; HEAT: 5486 ± 6229 IU L^−1^, 38‐fold increase, *P* < 0.01) as did serum myoglobin (NON: 362 ± 483; HEAT: 355 ± 373 μg L^−1^, 13‐fold increase, *P* < 0.03). Plasma HSP 70 was higher (*P* < 0.02) in HEAT after 120‐h of recovery. There were no differences between treatments for plasma HSP27 and interleukins 1*β*, 6, and 10. The results indicate that >40°C muscle temperature does not alter skeletal muscle injury or functional impairments induced by novel ECC.

## Introduction

Physical damage to muscle fibers occurs when skeletal muscles are stressed by strenuous, unaccustomed exercise; manifestations of damage include delayed onset muscle soreness (DOMS) and prolonged losses in muscle strength and range of motion (Clarkson and Hubal 2002). Most of the work examining the effect of eccentrically biased exercise has been completed in individuals who were not hyperthermic and did not have elevated skeletal muscle temperatures indicative of high core temperatures. Thus, there is no clear link between increased muscle temperature and losses in muscle strength and DOMS. Epidemiological data indicate that novel exercise in relatively untrained individuals completed in a hot environment is associated with rhabdomyolysis (dark urine, extreme muscle soreness, elevated myoglobin in blood or urine) (Carter et al. 2005). Furthermore, there is a seasonal effect on injury rates such that in warmer months, higher environmental temperatures are associated with higher injury rates and incidence of rhabdomyolysis. Specifically, between 2004 and 2008 the pattern of exertional rhabdomyolysis followed a seasonal trend with nearly 75% of diagnoses occurring from May through September (Armed Forces Health Surveillance Center, 2009). While these data establish an association between environmental heat stress and rhabdomyolysis, little is known regarding the mechanism for this increased incidence. Assayag et al. (2010) and Horowitz (2014) suggest that at the beginning of heat exposure (first few days), cellular integrity is compromised leading to an inability to cope with novel stressors; certainly heat‐shock proteins (HSP) levels are lower (constituent and inducible) during the initial days of heat acclimation (McClung et al. 2008). Thus, a reduced availability of these molecular chaperones for repair might result in accentuated muscle damage and higher incidence of rhabdomyolysis following novel exercise occurring during the early stages of heat stress exposure.

Several studies have examined the possible impact of increased skeletal muscle temperatures on muscle injury (Evans et al. 2002; Brock Symons et al. 2004; Nosaka et al. 2004; Skurvydas et al. 2008). To control for possible confounding by exercise, these studies used microwave diathermy, ultrasound, or warm‐water immersion to locally heat muscle. Overall, these studies found no or little effect from increased muscle temperatures (mean, 38.4°C) on functional and clinical outcomes induced by eccentric exercise, contrary to what is stated about the benefit of a “warm‐up” before exercise. However, it has been well documented that during intermittent (Drust et al. 2005; Morris et al. 2005) or continuous (Febbraio et al. 1994a,b) exercise in 33–40°C ambient temperatures, internal muscle temperature may exceed 40°C. There are no prospective studies that have identified whether acute increases in muscle temperature above 40°C increase susceptibility to muscle injury in a human model. This 40°C muscle temperature threshold is associated with leakage from the sarcoplasmic reticulum (van der Poel and Stephenson 2007) and with core temperatures (39.6–39.7°C; refs (Febbraio et al. 1994a,b) associated with heat exhaustion/injury (Sawka and O'Connor 2015). Furthermore, during physical exercise active muscle often increases by >2°C and this may help mediate mitochondrial damage as release of DNA fragments are associated with systemic inflammatory responses and heat stroke (Sawka et al. 2011).

This study examined if high skeletal muscle temperatures (>40°C) augments skeletal muscle injury induced by eccentric exercise. We also explored the effects of heating on various circulating cytokines and HSPs related to muscle adaptation and remodeling. It was hypothesized that local muscle heating applied just before exercise will cause greater skeletal muscle damage compared to eccentric exercise performed without muscle heating. We used a controlled model of local muscle heating and muscle damage to characterize the effect of high muscle temperatures on muscle damage and commenced eccentric exercise as soon as the heating protocol was completed.

## Methods

### Subjects

Five men and three women volunteers provided written informed consent to participate in this study, which was approved by the Scientific and Human Use Review Boards of the U.S. Army Research Institute of Environmental Medicine and the U.S. Army Medical Research and Materiel Command. The subjects volunteered after being fully informed of the requirements and risks associated with the research. Investigators adhered to Army Regulation 70–25 and U.S. Army Medical Research and Materiel Command Regulation 70–25 on the use of volunteers in research. Subject characteristics (mean, SD) were: age, 22.5 ± 4.1 year; height, 169.5 ± 10.8 cm; and body mass, 76.2 ± 12.6 kg. The subjects for this study were not heat acclimatized as the experiments were conducted in the autumn and winter months and abstained from any exercise‐heat stress, but they were active and exercised aerobically 3–5 times per week. The volunteers were not engaged in any resistance training for the upper body within the last 6 months.

### Experimental design

A crossover design was used with each volunteer serving as their own control. We also controlled for time of day and hydration Volunteers were randomly assigned to a specific eccentric exercise treatment order for the first test (heated (HEAT) or nonheated bicep muscle (NON); left or right arm). There was a 6‐week washout period between eccentric exercise trials. Following the washout period, subjects eccentrically exercised the opposite arm under the second treatment condition. Subjects had no caffeine or alcohol 12 and 48‐h before testing, respectively. As well, subjects were also tested in a euhydrated state, confirmed by ensuring the urine specific gravity was below 1.020 for a first morning void.

### Eccentric exercise protocol

Eccentric exercise consisted of two bouts of 24 maximal eccentric actions (muscle lengthening contractions) of the elbow flexor muscles in the heated or nonheated arm. The subject was seated at a modified preacher curl bench with a bar attached to the exercise lever. The subject attempted to curl the lever upwards from a 90° flexed position while a force was applied against the attachment bar to resist the subject's motion and extend the arm, producing a maximal eccentric contraction. Each exercise contraction lasted for 3‐sec in duration and was repeated every 5‐sec. There was a 2 min rest period between the first and second eccentric exercise bout. Total exercise time was approximately 10 min.

### Muscle heating

Immediately before the eccentric exercise, 100 Watts of shortwave diathermy (AutoTherm 390, Mettler Electronics Corp., Anaheim, CA) was applied for 15 min to the biceps muscle. The volunteer was supine when diathermy was applied. The drum was placed directly over the largest area of the bicep; a paper towel was placed between the drum head and the skin. As well, in between eccentric exercise bouts, diathermy was applied for 2 min to ensure that muscle temperatures remained high during the second bout of eccentric contractions. Shortwave diathermy uses condenser and electromagnetic inductive field coils to deliver short, high‐energy pulses that heat tissue underneath the shortwave applicators (via a capacitive method to areas of low blood circulation or inductive method to heat deeper areas of high blood circulation, i.e., muscle). Shortwave diathermy delivers energy in the radio band of 27.12 MHz. Our pilot data (*n* = 4 tests on three individuals who were not in the study) demonstrated that a continuous power output of 100 Watts given for 15 min increased the biceps muscle temperature to 40.3°C at a depth of 2.5 cm (individual bicep muscle temperatures were 41.5, 39.0, 40.2, and 40.6°C at the completion of diathermy in these four pilot tests). Muscle temperature remained elevated for approximately 5 min post heating. For these pilot experiments, the biceps was first anesthetized with 3 mL of 1% lidocaine and an 18‐gauge catheter inserted into the muscle using sterile techniques. The pilot volunteer was then given the diathermy treatment. Immediately following the 15‐min heating period with the shortwave diathermy, a temperature probe was inserted into the catheter and muscle temperature was measured. Muscle temperature was not measured during the actual experiment because the muscle damage from the insertion of the catheter/temperature probe would have confounded the results from the two trials.

## Measurements

Our measurement procedures were consistent with those previously used to examine the impact of eccentrically biased exercise on functional outcomes and surrogates of muscle damage (Clarkson et al. 1992; Nosaka and Clarkson 1997; Nosaka et al. 2002).

### Isometric strength

Maximal voluntary isometric contraction (MVC) strength was measured by performing three maximal contractions (3‐sec in duration) with the arm flexed at 90 degrees (Jackson Strength Evaluation System, Lafayette Instrument Corp., Lafayette, IN). The subject sat on the isometric preacher curl machine, with straight legs and both heels on the floor to prevent extraneous movement/bracing that would influence the strength measures. Subjects were asked to perform the MVC three times. One minute rest was given after each test.

### Sorenes

Soreness was assessed as the subjects performed a biceps curl using a low weight (0.45 kg for subjects <68.2 kg and 0.9 kg for subjects >68.2 kg) by extending their arm straight‐out in front of them and then returning their arm to their side in a flexed position. Subjects recorded the peak soreness of their arm muscle on a Visual Analog Scale where the left anchor of “no soreness” was at 0 mm and “very, very sore” was anchored at 100 mm. Subjects were instructed to draw a vertical line bisecting the point on the line that best described their muscle soreness while doing the contraction.

### Range of motion and circumference

Range of motion (ROM) was assessed, using a goniometer, in two arm positions, relaxed and flexed. For the relaxed ROM, the subject hung their arm passively by their side. A semipermanent marker was used to mark the middle of the wrist, the lateral epicondyle of the humerus, and the acromion process of the scapula to ensure reproducibility in subsequent measurements. The middle of the goniometer was placed over the mark on the elbow. One end of the goniometer was aligned with the shoulder mark and the other over the wrist mark. For the flexed ROM measure, the volunteer flexed their bicep as much as possible and the angle was measured through the same points as the relaxed ROM. Biceps muscle circumference was measured with a tape measure.

### Blood measures

Blood was sampled from a butterfly catheter inserted into a superficial forearm vein. Blood was analyzed, in duplicate, for serum creatine kinase, myoglobin, creatinine, blood urea nitrogen (BUN), aspartate aminotransferase (AST), alanine aminotransferase (ALT), and serum alkaline phosphate (ALP) using an automated clinical chemistry analyzer (Siemens Dimension Xp and Plus Integrated Chemistry System). Blood was collected in serum separation tubes following 30‐min of clotting. The tubes were then centrifuged at 4°C at 2500 rpm for 10‐min. Also analyzed, in duplicate, were circulating markers of the heat stress response (HSP 27, 70) and inflammation (cytokines, IL‐1 *β*, IL‐6, IL‐10). Blood for the HSP analysis was collected in serum separation tubes and serum was obtained in a similar manner as detailed above for the clinical measurements. The tubes were then centrifuged at 4°C at 2500 rpm for 10‐min.The HSP's were assayed by enzyme‐linked immunosorbent assay (ELISA) on a Dynex DS‐2 with kits from Enzo Life Sciences (Plymouth Meeting, PA); the kit number for HSP27 was ADI‐EKS‐500 and HSP70 was ADI‐EKS‐715. The intraassay % coefficient of variation's (%CV) were 9.6 and 10.2%, for HSP27 and HSP70, respectively. Blood for interleukin analysis was collected in EDTA tubes and then centrifuged at 22°C at 1000 rpm for 15‐min. Plasma IL‐1*β*, IL‐6, and IL‐10 were all run on the Luminex Magpix Multiplex System, (kit HCYTOMAG‐60K, Millipore, Billerica, MA). Intraassay %CV's were 9.4, 7.5, and 5.2% for IL‐1 *β*, IL‐6, and IL‐10, respectively.

### Statistical analyses

Data were analyzed using a two‐way analysis of variance (ANOVA) with repeated measurements (treatment × time). Outcome measurements were obtained before eccentric exercise (PRE), immediately postexercise (POST) and at 6, 24, 48, 72, 120, and 240 h post‐ECC. Primary outcome variables included strength, soreness, range of motion, and blood variables. Tukey's HSD procedure was performed post hoc when significant main effects or interactions were identified using repeated measures ANOVA. Statistical significance was set at *P* < 0.05. A power analysis (*β *= 0.80 at alpha of 0.05) on the change in % MVC, considered the most important outcome measure, indicated that seven volunteers would be needed to detect differences at the 48‐h post‐ECC time period.

## Results

### MVC, soreness, ROM, circumference

**Figure 1:**
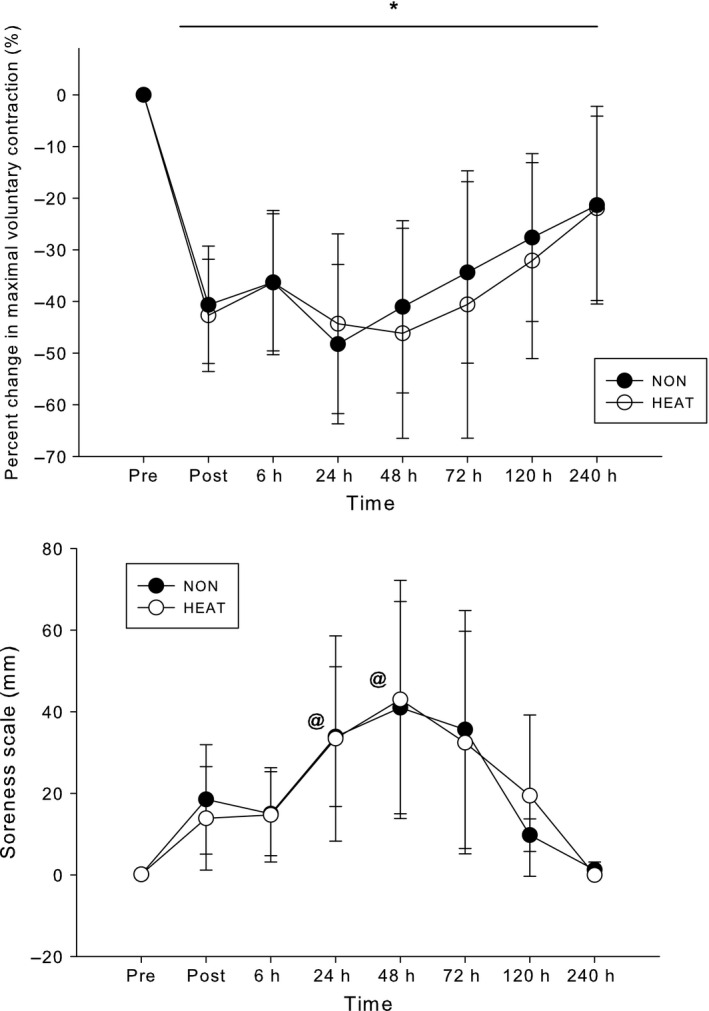
Change in maximal voluntary contraction and soreness versus time following no heat (NON) and diathermy heat (HEAT) trials. *significant difference (*P *< 0.0001) from Pre; @, significantly higher versus Pre, Post, 6‐h, 120‐h, and 240‐h (*P *< 0.05). There were no differences between NON and HEAT.

**Figure 2:**
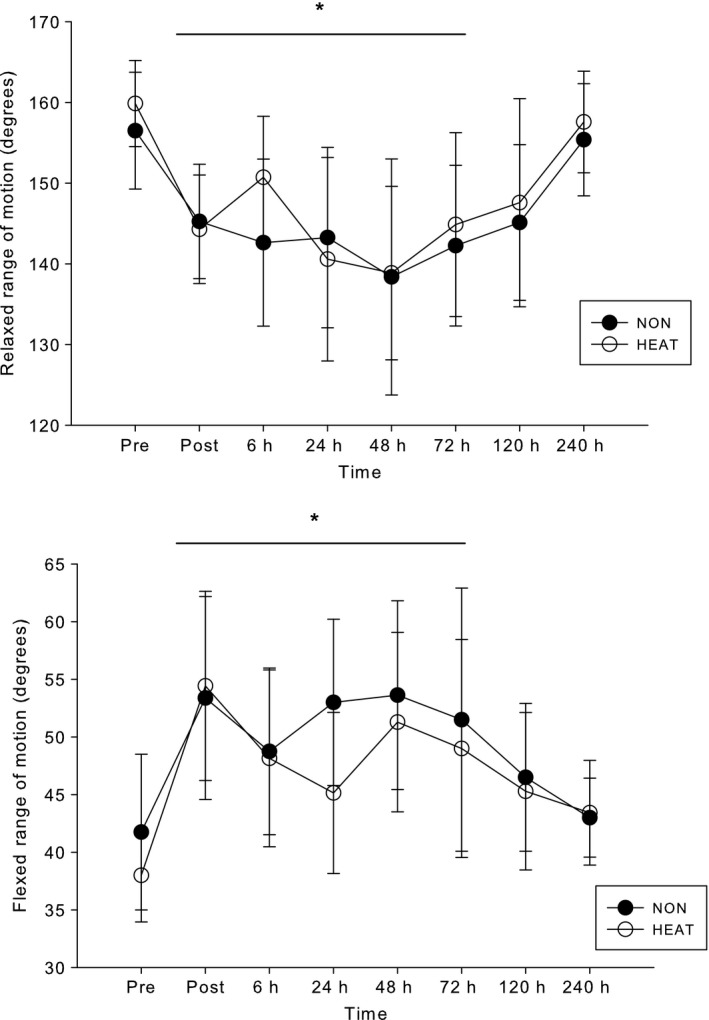
Relaxed and flexed range of motion versus time following no heat (NON) and diathermy heat (HEAT) trials. *significantly lower (*P *< 0.003) versus Pre. There were no differences between NON and HEAT.

Figure 1 presents the isometric strength and muscle soreness scale results. Subjects had a 46–48% decline in strength following the eccentric exercise bouts (*P* < 0.001), but there was no difference between NON and HEAT (absolute baseline MVC was 41.9 ± 16.4 kg). MVC recovered from its nadir at 24–48‐h, but was still lower than the baseline value 240‐h after ECC (*P* < 0.001). Concurrently, there was a 42‐point rise in the muscle soreness rating 2 days after ECC (*P* < 0.05); by 240‐h, soreness returned to PRE values. These measurements were not different between the HEAT and NON trials. The relaxed ROM decreased (*P* < 0.001)and the flexed ROM increased (*P* < 0.003), respectively (Fig 2), but there were no differences between HEAT and NON (*P* > 0.05). Mean bicep circumference was ~ 4% higher for the 120‐h period after ECC (*P* = 0.04), with no difference between the HEAT and NON trials (baseline circumference was 31.3 ± 2.6 mm).

### Creatine kinase, myoglobin, and liver enzymes

**Figure 3:**
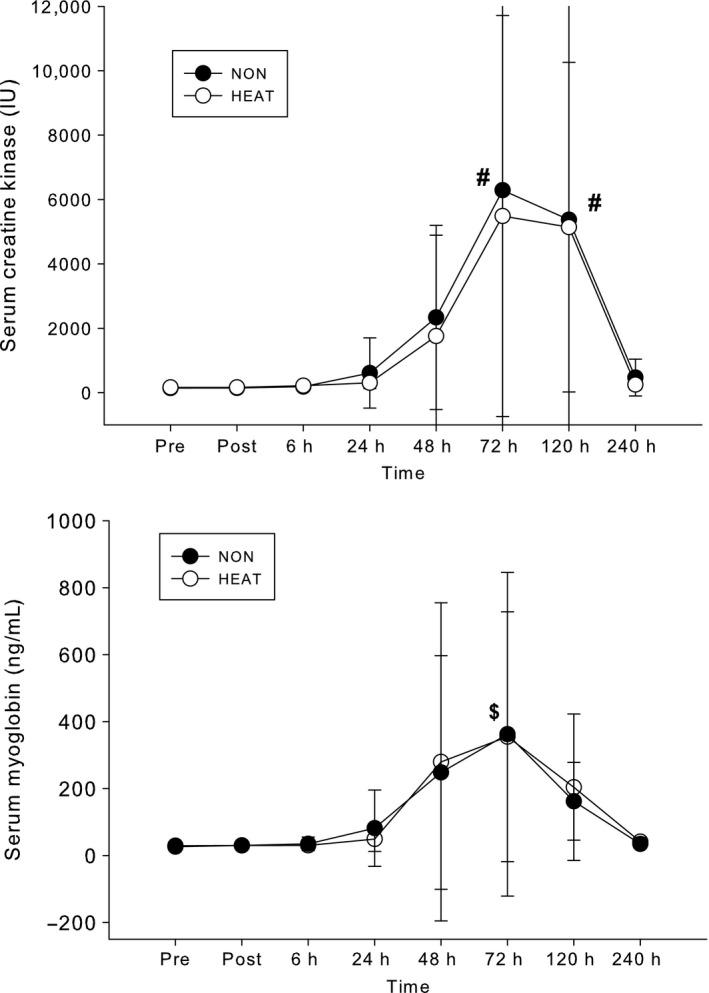
Serum creatine kinase and myoglobin versus time following no heat (NON) and diathermy heat (HEAT) trials. ^#^72‐h and 120‐h significantly higher (*P *< 0.01) versus all other time points; $, 72‐h significantly higher (*P *< 0.003) than Pre, Post, 6‐h, 24‐h, and 240‐h. There were no differences between NON and HEAT.

Blood markers of muscle damage are presented in Figure 3. ECC caused a large increase in serum CK (increased 3200–4325%) and serum myoglobin (increased 1200%) levels. Serum CK was elevated at 72‐h and 120‐h (*P* < 0.01), compared to all other time points; myoglobin was significantly elevated at 48‐h (*P* < 0.03), compared to PRE. There were no differences in CK or myoglobin between the HEAT and NON trials. Markers of liver and kidney function are presented in Table 1. Two liver markers, AST and ALT, were significantly elevated 3–5 days after ECC (*P* < 0.03), peaking at levels above clinical normative values, and then returned to baseline levels after 10 days of recovery. No differences were noted between treatments. The other markers, ALP and BUN, did not change over time and were not different between NON and HEAT.

**Table 1:** Liver marker concentrations over time following eccentric exercise subsequent to no heating (NON) and heating (HEAT) of the elbow flexor

	Time	Pre	Post	6‐h	24‐h	48‐h	72‐h	120‐h	240‐h
	Trial								
AST (U L^−1^)	NON	24.9 ± 5.4	26.2 ± 5.3	28.0 ± 8.8	31.3 ± 10.1	60.4 ± 43.5	106.0 ± 135.9^a^	120.1 ± 135.5^a^	38.9 ± 24.7
	HEAT	27.2 ± 8.2	27.3 ± 8.9	25.1 ± 7.4	27.7 ± 7.4	46.5 ± 49.6	111.8 ± 110.6^a^	134.2 ± 97.5^a^	29.8 ± 9.0
ALT (U L^−1^)	NON	36.2 ± 17.0	36.4 ± 16.8	41.9 ± 21.0	39.0 ± 18.1	49.3 ± 26.8	53.5 ± 34.6^a^	61.5 ± 43.1^a^	48.0 ± 25.4
	HEAT	35.3 ± 18.3	35.6 ± 18.2	27.1 ± 10.7	33.9 ± 18.4	30.9 ± 17.1	51.8 ± 36.0^a^	68.5 ± 39.9^a^	40.0 ± 21.3
ALP (U L^−1^)	NON	91.4 ± 25.3	95.8 ± 26.5	93.4 ± 26.3	94.7 ± 26.5	100.7 ± 25.0	95.3 ± 29.6	92.6 ± 23.3	93.9 ± 23.9
	HEAT	88.4 ± 20.8	89.6 ± 24.0	91.1 ± 20.1	94.3 ± 22.6	95.0 ± 21.0	95.1 ± 29.3	91.3 ± 24.6	91.4 ± 26.0
BUN (mg dL^−1^)	NON	12.7 ± 2.6	12.5 ± 2.5	14.8 ± 4.4	11.3 ± 4.1	13.1 ± 4.1	12.1 ± 3.1	11.7 ± 3.1	11.3 ± 2.8
	HEAT	11.8 ± 4.1	12.0 ± 4.1	12.8 ± 1.7	12.1 ± 3.8	9.8 ± 2.2	11.4 ± 2.6	10.9 ± 2.9	11.1 ± 1.2

a Denotes significant difference (*P *< 0.03) compared to Pre, Post, 6‐h, 24‐h, and 48‐h.

### Heat‐shock proteins and interleukins

Table 2 presents the heat shock and cytokine response. For HSP 27, there were no differences between NON and HEAT after ECC. Plasma HSP 27 levels remained at baseline values through 48‐h and then peaked 3–5 days after ECC (*P* < 0.05 between 24‐h and 72‐h and *P* = 0.055 between 24‐h and 120‐h). Otherwise there were no other differences in HSP 27. HSP 70 was different at 120‐h between the two trials, with plasma values higher in the HEAT trial versus NON (*P* < 0.05). Also, there was a main effect observed between trials with the HEAT trial exhibiting higher overall HSP 70 levels compared to NON. There were no differences between trials for plasma IL‐10, IL‐1 *β*, and IL‐6 (*n* = 4). Furthermore, there were no changes over time in these cytokines.

**Table 2:** Plasma heat‐shock protein and interleukin concentrations over time following eccentric exercise subsequent to no heating (NON) and heating (HEAT) of the elbow flexor

	Time	Pre	Post	6‐h	24‐h	48‐h	72‐h	120‐h	240‐h
	Trial								
HSP27 (ng mL^−1^)	NON	2.91 ± 1.21	4.29 ± 2.22	2.59 ± 1.44	2.51 ± 1.65	3.46 ± 2.44	5.77 ± 3.78	5.29 ± 3.23	2.68 ± 1.41
	HEAT	3.00 ± 2.89	3.76 ± 4.15	4.17 ± 2.60	2.48 ± 1.58	2.74 ± 1.68	5.66 ± 4.17^a^	5.92 ± 4.45	2.73 ± 2.02
HSP70 (mg mL^−1^)	NON	0.34 ± 0.09	0.36 ± 0.10	0.29 ± 0.10	0.27 ± 0.11	0.31 ± 0.10	0.35 ± 0.10	0.28 ± 0.11	0.27 ± 0.10
	HEAT^c^	0.33 ± 0.11	0.34 ± 0.10	0.35 ± 0.12	0.29 ± 0.10	0.31 ± 0.09	0.34 ± 0.08	0.37 ± 0.12^b^	0.31 ± 0.07
IL10 (pg mL^−1^)	NON	3.17 ± 1.50	4.20 ± 2.20	3.71 ± 2.33	3.44 ± 1.85	3.17 ± 1.44	3.10 ± 2.44	4.24 ± 2.51	2.87 ± 1.67
	HEAT	2.51 ± 1.37	2.91 ± 1.91	2.79 ± 1.80	2.91 ± 1.73	2.96 ± 1.99	3.07 ± 1.49	2.89 ± 1.94	2.58 ± 2.08
IL1*β* (pg mL^−1^)	NON	0.78 ± 0.50	1.10 ± 0.41	0.60 ± 0.45	0.93 ± 0.83	0.85 ± 0.59	0.50 ± 0.14	1.10 ± 0.56	0.98 ± 0.56
	HEAT	0.70 ± 0.38	0.55 ± 0.26	0.68 ± 0.28	0.48 ± 0.33	0.70 ± 0.23	0.65 ± 0.37	0.75 ± 0.37	0.57 ± 0.32
IL6 (pg mL^−1^)	NON	0.55 ± 0.07	1.07 ± 1.10	1.47 ± 1.01	0.83 ± 0.71	0.95 ± 0.07	0.90 ± 0.57	1.23 ± 0.80	0.63 ± 0.46
	HEAT	0.70 ± 0.05	0.75 ± 0.35	1.20 ± 0.66	0.67 ± 0.60	0.77 ± 0.42	2.90 ± 2.55	1.17 ± 0.78	0.70 ± 0.85

HSP27 (*n* = 8); HSP70 (*n* = 7); IL10 (*n* = 8); IL1*β* (*n* = 4); IL6 (n‐3).

a Denotes significant difference (*P *< 0.05) between 24‐h and 72‐h for HSP27.

b Denotes significant difference (*P *< 0.02) between NON and HEAT for HSP70 at specified time.

c Denotes significant main effect (*P* < 0.05), HEAT > NON for HSP70.

## Discussion

This study was the first to experimentally evaluate the acute effects of muscle temperature >40°C on muscle damage and function following eccentrically biased exercise. This temperature level was chosen as muscles can get this hot during exercise (Febbraio et al. 1994a,b; Drust et al. 2005; Morris et al. 2005), and the previous studies did not heat muscle to this temperature. Although diathermy was applied to elevate skeletal muscles to ~40°C before exercise, it is likely that the eccentric contractions further increased skeletal muscle temperature. Time of day and hydration were controlled, and the study utilized a crossover design so that volunteers served as their own controls to reduce intersubject variability. The principle findings from this study were that eccentric exercise following local heating does not alter most functional, subjective, and blood indices of muscle damage. HSP 70 levels were higher across time in the HEAT trial, but the inflammatory cytokine response was not affected by temperature. These data suggest that acute local muscle heating does not increase the risk of heat‐related rhabdomyolysis.

Eccentric exercise commonly causes a 20–55% acute decline in voluntary muscle force development, which resolves gradually over 1–2 weeks (Clarkson et al. 1992; Hubal et al. 2007). Similar results were observed in this study. This decline in force and subsequent recovery was not impacted by the muscle temperature during inducement of the muscle damage. Similarly, other markers of DOMS such as soreness, bicep circumference, creatine kinase, and myoglobin (Clarkson and Tremblay 1988; Clarkson et al. 1992; Clarkson and Hubal 2002) were also unaffected by an increase in local muscle temperature.

Our findings agree with previous studies that increased muscle temperature before eccentric exercise has no effect on muscle strength and markers of damage. Evans et al. (2002), in four subjects, raised the biceps brachii temperature by ~ 3.5°C (absolute temperature ~ 38.5°C) with short‐wave diathermy before performing maximal eccentric contractions of the muscle group. Functional (maximal voluntary contraction) and subjective (soreness) outcomes were followed up for up to 6‐day post eccentric exercise. These responses were compared to separate control subjects (*n* = 10) who did not have biceps heating. No differences were observed between treatments and the interindividual variability was large. Nosaka et al. (2004) also raised muscle temperature in the biceps brachii by 3°C using microwaves (to ~ 37.5°C) in 10 subjects (who served as their own controls) and observed no effect on responses to subsequent eccentric exercise. Brock Symons et al. (2004) also found no effect of raising biceps temperature by 2°C using ultrasound. In contrast, Skurvydas et al. (2008) used water immersion (bath temperature of 44°C) to raise vastus lateralis temperatures to 39.4°C. Subsequent stretch‐shortening exercise (jumping from a platform, bending the knees to a 90° angle and then jumping again) was then performed with outcomes followed up for 3‐day postjumping. Markers of muscle damage (plasma creatine kinase, subjective ratings of soreness) were attenuated in the muscle heating treatment and there was a significant, albeit very small, improvement in MVC at one recovery time point (48‐h) after eccentric exercise. Differences in outcome measurements following heat treatment may be different in Skurvydas et al. (2008) due to their use of plyometrically based exercise, compared to the other studies using eccentric‐only exercise. Overall, in summary, the studies suggest that heating muscle acutely over relatively short durations before eccentric exercise has no beneficial or harmful effect on muscle function or markers of muscle damage.

There is some evidence that skeletal muscle injury may be protected by muscle heating 1 day before initiation of eccentric exercise. Nosaka et al. (2007) found that a 20‐min exposure to a microwave, which increased muscle temperature to ~ 41°C, protected against subsequent eccentric‐induced muscle injury the next day. Losses in maximal isometric strength, muscle range of motion, and subjective muscle soreness were all attenuated following heat treatment. This response could potentially represent an early adaptation response (increased HSP response on second heat exposure) as heating occurred 1 day before eccentric exercise and protein synthesis induced by heat exposure may be preventing the eccentric exercise‐induced muscle damage (Koh 2002; McArdle et al. 2004; Ogura et al. 2007, 2008). Nosaka stated in his 2007 paper (Nosaka et al. 2007) that “it would be interesting to examine whether or not microwave diathermy treatment >40°C implemented immediately before eccentric exercise would induce a similar protective effect.” This study's findings suggest that heating applied acutely before eccentric exercise does not provide a protective effect as observed in Nosaka et al. (2007), but the immediate application before exercise does not provide sufficient time for HSP induction and thus protective functions.

Several clinical markers (AST and ALT) were elevated following ECC but were not affected by prior muscle heating. Similarly, Kanda et al. (2014) observed a 550% increase in AST after eccentrically biased exercise in the calf. The functional significance of these increases in serum transaminases is unclear as it may not be indicative of a pathophysiological condition and thus not a sensitive biomarker with clinical significance. For example, several papers report high transaminase levels with idiopathic inflammatory myopathy (Mathur et al. 2014) and rhabdomyolysis (Weibrecht et al. 2010) that do not correlate with liver injury. In fact, in those studies, these liver biomarkers tend to rise and decline in conjunction with CK levels, similar to what was observed for AST and ALT in this study.

Plasma HSP70 was elevated in the HEAT trial 5 days after the treatment, with the later observation likely the result of the extra heat exposure. Others have also observed increases in muscle HSP 70 levels following elevated muscle temperature induced through passive means (Ogura et al. 2007), although this finding is not universal (Morton et al. 2007; Vardiman et al. 2015). The significance of this small increase in plasma HSP70 is unclear. Higher HSP70 levels could potentially be protective against injury and loss of functional deficits, but at the time point where HSP70 levels were significantly higher in HEAT, compared to NON (5‐day post‐ECC), no difference was observed between trials in MVC or any other muscle damage marker.

Prior muscle heating had no effect on the small HSP, HSP 27. However, ECC led to a delayed increase in plasma HSP 27 levels; values peaked 3–5 days following ECC. Previous studies (Thompson et al. 2001; Paulsen et al. 2007; Frankenberg et al. 2014) have documented a rise in muscle HSP27 following ECC, with these small HSPs migrating from the cytosol to the cytoskeleton (Frankenberg et al. 2014) and acting as a stabilizer by binding to cytoskeletal/myofibrillar proteins (Paulsen et al. 2007). Interestingly, the cytosolic HSP27 response is positively related to muscle function, that is, the greater the loss in muscle strength, the greater the cytosolic loss of protein (Paulsen et al. 2007). Plasma HSP27, although not indicative of cytosol levels, peaked ~ 24‐h after strength reached its nadir, possibly indicating heat shock protein loss from the muscle. The correlation between the 48‐h loss in strength (% MVC change) and the 72‐h plasma HSP27 values demonstrated a moderate relationship (*r* = 0.59), possibly reflecting this protein loss and its linkage to the observed strength changes.

No changes were observed either across time or between treatments for the pro‐ and anti‐inflammatory cytokines IL‐1 *β*, IL‐10, and IL‐6. Hirose et al. (2004) also found that ECC of the elbow flexors had no effect on plasma IL‐10 and IL‐6. One possible reason is that peak values were missed with the timing of blood samples. For example, IL‐6 peaks 2‐8 h after ECC and is back to normal by 24‐h (Bruunsgaard et al. 1997; Willoughby et al. 2003; Miles et al. 2008). Therefore, even though IL‐6 was postulated to be related to muscle damage (Bruunsgaard et al. 1997), the response is quite transient. A comprehensive review by Pedersen (Pedersen 2013) suggests that ECC does not increase IL‐6 levels above that observed with concentric exercise.

In conclusion, our results indicate that experimentally induced muscle temperatures above 40°C, muscle temperatures that can be achieved during high intensity exercise, neither accentuates nor reduces skeletal muscle injury or functional impairments induced by novel eccentric exercise. It is important to note that 40°C was the initial muscle temperature and that eccentric exercise would further elevate the muscle temperature. This suggests that high muscle temperature alone might not be responsible for the observation that novel exercise performed in a hot environment increases susceptibility to rhabdomyolysis in unacclimatized athletes and Soldiers.

## Conflict of Interest

The authors have no conflict of interest to declare.
